# Invasive cystic hypersecretory carcinoma of the breast

**DOI:** 10.4322/acr.2021.375

**Published:** 2022-04-28

**Authors:** Srilata Chitti, Sunayana Misra, Arvind Ahuja, Nikhil Gupta, Raghav Yelamanchi

**Affiliations:** 1 Atal Bihari Vajpayee Institute of Medical Sciences and Dr RML Hospital, Department of Pathology, New Delhi, India; 2 Atal Bihari Vajpayee Institute of Medical Sciences and Dr RML Hospital, Department of Surgery, New Delhi, India

**Keywords:** Breast, Mammography, Breast cyst, Breast Neoplasms

## Abstract

Cystic hypersecretory carcinoma (CHC) of the breast is a rare variant of ductal carcinoma, characterized by variably sized cysts lined by micropapillary fronds to proliferative pseudostratified columnar epithelium. It includes a spectrum of morphological features ranging from clearly benign cystic hypersecretory hyperplasia (CHH), CHH with atypia to invasive CHC. Only 20 cases of invasive CHC have been reported to date. We report a case of a 49-year-old female who presented with a palpable breast lump and nipple discharge. Gross examination showed variable-sized cysts lined by solid grey white tumors. On microscopic examination, cysts were lined by micropapillary fronds with eosinophilic colloid-like secretion with a focus of invasion. A diagnosis of invasive CHC was made. Since there are limited case reports, our understanding of its biological behavior, prognostic factors, and genetic basis is limited.

## INTRODUCTION

Cystic hypersecretory carcinoma (CHC) is a rare subtype of breast ductal carcinoma, which was first described by Rosen and Scott^1^ in 1984. It is characterized by the presence of cysts of varying sizes filled with amorphous acellular colloid-like eosinophilic material.^1^ Microscopically, these cysts are lined by micropapillary to the pseudostratified epithelium. CHC is a distinctive variant of ductal carcinoma in situ (DCIS) that arises in a background of cystic hypersecretory hyperplasia (CHH) and/ or CHH with atypia.^2^ The morphological features of cystic hypersecretory lesions of the breast range from benign (CHH) to a combination of the benign and atypical epithelium (CHH with atypia) to the malignant epithelium. CHH shows cysts lined by a single layer of cuboidal to columnar cells with bland nuclei and inconspicuous cytoplasm. CHH with atypia has cysts with columnar cells, which show atypia and epithelial cell crowding, hyperchromatic nuclei, and few nucleoli. The characteristic findings of invasive CHC are the formation of dilated ducts lined by pseudostratified to the micropapillary epithelium. Cells are densely packed, with overlapping hyperchromatic nuclei and sparse cytoplasm containing intermediate to high-grade nuclei.^1^ Only 20% of CHC have been identified as invasive. In this setting, the invasive component is usually poorly-differentiated with the lack of secretory features.^3^ Twenty cases of invasive CHC have been reported to date.^4^ Due to its rarity, CHC was not included in the 2019 WHO classification of breast tumors.

We present a case of invasive CHC, the twenty-first reported case based on an extensive review of literature.^2^^-^16 We searched PUBMED for the keywords “CHH”, “CHC”, “CHC invasive”. A total of 10 case reports of invasive CHC were retrieved. Five case series of cystic hypersecretory lesions were retrieved, of which 8 cases showed invasive CHC and 2 cases showed microinvasive CHC (Table 1).^2^^-^16 The purpose of this report is to raise awareness of this rare subtype of breast carcinoma to avoid misdiagnosis as a benign apocrine lesion, DCIS, or invasive duct carcinoma (IDC).

**Table 1:** Summary of previously reported invasive CHC cases

	**Age (y)/Gender**	**Type**	**Lymph node**	**ER/PR status**	**Her2/neu**
Rosen and Scott^1^	52/F	invasive	N1	Positive	NA
	47/F	invasive	N0	NA	NA
	62/F	invasive	N1	NA	NA
Guerry et al.^6^	NA/F	invasive	N1	NA	NA
	NA/F	invasive	metastatic	NA	NA
Adams and Lacey^10^	70/F	micro invasive	N0	Negative/positive	NA
	37/F	invasive	N0	NA	
Kim et al.^11^	37/F	invasive	N0	NA	NA
Herrmann et al.^12^	49/F	invasive	N0	Positive/Positive	NA
Lee and Lee^13^	45/F	invasive	N0	Negative/negative	NA
Shin and Rosen^2^	42/F	invasive	N (micro)	NA	NA
Skalova et al.^7^	66/F	invasive	NA	Positive/Positive	NA
Chen and Kan^14^	44/F	Microinvasive	NA	Positive/Positive	NA
Song et al.^15^	43/F	invasive	NA	NA	Positive
D’Alfonso et al.^5^	62/F	Microinvasive	NA	Positive/Positive	NA
Bi et al.^16^	37/F	invasive	N1	NA	Positive
	46/F	invasive	NA	NA	Positive
Gupta et al.^9^	57/F	invasive	N0	Negative/Negative	NA
Sahoo et al.^8^	32/F	invasive	N1	Negative/Negative	Positive
Sun et al.^4^	63/F	invasive	N1	Negative/Negative	Positive
Our case	49/F	invasive	N0	Negative/Negative	Positive

F= female; NA= not available; y= years.

## CASE REPORT

**Figure 1:**
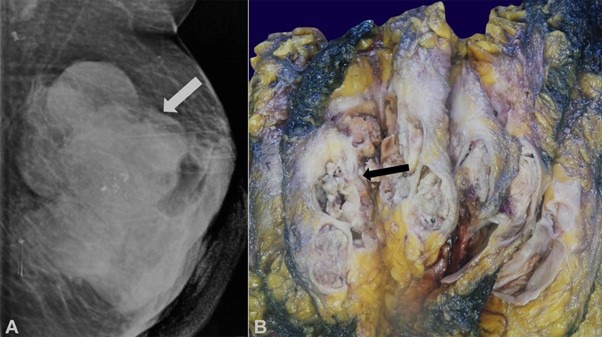
**A –** Mammography showing an ill-defined right breast sub-aerolar mass with eccentric lucent areas (craniocaudal view); **B –** Gross examination revealed a grey white tumor with multiple variably sized cysts containing friable mucoid material.

A 49-year-old female presented with the complaint of painful swelling in the right breast for six months. The swelling developed gradually and was associated with nipple discharge. On examination, an ill-defined mass was palpated in the upper outer quadrant of the right breast. The skin surface was blanched and erythematous. The contralateral breast was normal, no axillary lymph nodes were detected on palpation. Ultrasonography (USG) revealed a large heterogeneous, hypoechoic collection with interval septation and lobulation, as well as peripheral vascularity, involving all quadrants of breast and retroaleolar region along with overlying skin thickening, as well as subcutaneous edema. Contralateral breast tissue was found to have normal fibrous glandular tissue. An impression of breast abscess was given. Mammography revealed an ill-defined mass with irregular contours and eccentric lucent areas, as well as soft tissue swelling in the upper and central subareolar regions around the nipple (Figure 1A). The left breast appeared to be normal. No evidence of lymph node involvement was reported. Fine needle aspiration cytology (FNAC) was performed from the right breast mass, revealing inflammatory cells, cystic macrophages, and degenerated cells in a proteinaceous background. A trucut biopsy performed from the breast mass showed dense abundant eosinophilic, acellular proteinaceous material, foamy macrophages along with few degenerated cells. Repeat trucut biopsy revealed a few dyscohesive clusters of atypical cells along with similar proteinaceous material, raising suspicion of malignancy.

A simple mastectomy was performed, which on gross examination revealed a grey-white tumor measuring 6.5×4×4 cm occupying the right upper and lower quadrants as well as the retroaereolar region (Figure 1B).

**Figure 2:**
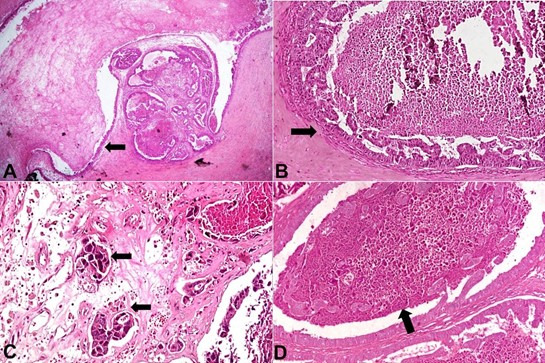
Photomicrographs of the tumor. **A –** Variable sized cysts filled with thyroid colloid like eosinophilic secretions (H&E; x200); **B –** Cysts lined by micropapillary fronds with high nuclear grade (H&E; x200); **C –** Invasive component composed of nests of tumor cells invading stroma (H&E; x400); **D –** Secretory material is positive for PAS (PAS; x200).

Upon serial slicing the tumor, variably-sized cysts ranging from 1 to 2.5 cm were identified, containing thick gelatinous secretions (Figure 2A). The cysts’ walls were grey-white, fleshy to firm. Scattered calcified areas were also observed. Since no axillary lymph nodes were palpated, no sentinel lymph nodes were biopsied. Microscopy revealed variably sized cysts lined by flattened cuboidal epithelium to tall micropapillary fronds in some areas, with moderate nuclear grade (Figure 2B). The cysts were filled with acellular amorphous, eosinophilic, colloid-like material and foamy macrophages. An invasive focus consisting of nests of irregular tumor cells, infiltrating the cyst wall was identified (Figure 2C). Additionally, a DCIS component was identified that showed solid, micropapillary, and comedo patterns. The secretion was positive for Periodic acid Schiff (PAS) (Figure 2D).

**Figure 3:**
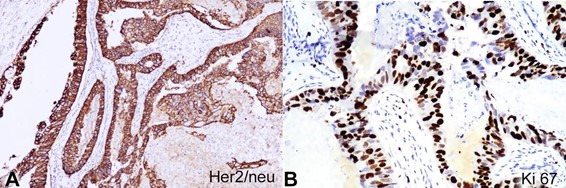
Photomicrographs of the tumor. **A –** Membranous positive for HER2/neu (in tumor cells (x400) ASCO/CAP guidelines IHC score 3+; **B –** Positive Ki 67 expression in tumor cells (x400).

The tumor was estrogen receptor (ER), progesterone receptor (PR) negative, and HER2/neu positive (ASCO/CAP guidelines IHC score 3+), (Figure 3A), with a Ki-67 index of 40-45% (Figure 3B). A diagnosis of invasive CHC was rendered. A right axillary node dissection was carried out; no tumor metastasis was detected. At 6 months follow-up, the patient has received 8 cycles of chemotherapy (Adriamycin, cyclophosphamide, paclitaxel) and is disease-free. The patient is also on 3 weekly Trastuzumab injections to be continued for a year.

## DISCUSSION

CHC is a rare subtype of ductal carcinoma characterized by the presence of luminal secretions resembling thyroid colloid.^5^ Guerry et al.^6^ described the entire spectrum of cystic hypersecretory lesions of the breast in 1988. The incidence of CHC is unknown due to its rarity and possible under-reporting and/or misclassification as a benign lesion or as ductal carcinoma in situ (DCIS).^1^

CHC has a similar age distribution to breast carcinoma. In a case series reported by D’Alfonso et al.,^5^ the patients’ ages ranged from 47 to 79 years. Another study by Skalova et al.,^7^ included five cases of CHC with patients’ ages ranging from 53 to 78 years. After extensively reviewing several case reports,^2^^-^16 it was observed that the youngest patient was 32 years old while the eldest was 79 years old.^2^^-^16 At the time of diagnosis, our patient was 49-years-old. Table 1 summarizes the previously reported cases of invasive CHC. Patients with CHC usually present with a palpable mass lesion with or without nipple discharge. D’Alfonso et al.^5^ noted that one of the ten patients presented with nipple discharge. Similarly, Sahoo et al.^8^ reported a case report in which the patient complained of nipple discharge along with a breast lump. Our patient also presented with breast swelling associated with nipple discharge. USG findings in various studies have been reported to be deceptively benign, most commonly being mistaken for fibrocystic changes or intraductal papilloma.^1^ USG findings in our case suggested a breast abscess. The results of mammography are variable. Mammography yields different findings; it usually shows increased breast density with trabecular thickening or may reveal a spiculated mass with calcification.^1^

Mammographic calcification and spiculations were observed in only three of ten cases reported by D’Alfonso et al.^5^ Microcalcification was not observed in any of the cases reported by Skalova et al.^7^ Although no calcification was visible on mammography in our case, scattered calcific areas were identified on gross examination and confirmed on histopathology. Thus as mentioned above, the radiographic appearance of CHC is quite variable, many times reported as benign, leading to misdiagnosis if the histopathology is not thoroughly performed.

FNAC is also usually inconclusive due to the secretion of copious and amorphous secretions. However, multiple passes taken, especially from solid areas/edge of the cysts may show atypical cells, raising the suspicion of malignancy.^1^ In the study by Skalova et al.,^7^ FNAC was performed in three (of five) cases with two revealing negative findings and one indicating malignancy. In our case, FNAC revealed inflammatory cells with a few degenerated cells in a blood background, similar to the findings of Sahoo et al.^8^ The trucut biopsy; however, it showed part of the cystic wall which was lined by atypical cells providing clues to raise the suspicion of malignancy. This, the onus lies on the pathologist to make an accurate, definitive diagnosis on histopathology as radiology and FNAC are often misleading.^8^

A larger excisional biopsy is required if the needle core biopsy shows features of CHH as it may have associated atypia, CHC, or invasive carcinoma. Extensive sampling is required to detect an invasive component in CHC. Biopsy taken from a non-representative area may miss the invasive focus. Excisional biopsy and FNAC were performed in the majority of cases reported in the literature. In the study by D’Alfonso et al.,^5^ nine of ten cases had excisional biopsies, and one case had needle core biopsy. FNAC and initial trucut biopsy were inconclusive in our case; however, subsequent attempts revealed few clusters of atypical cells. Still, the material obtained on biopsy was insufficient for proper classification of the lesion. A simple mastectomy was performed. The gross appearance of CHC is unique and shows multiple variably sized cysts with intraluminal mucoid to gelatinous secretions. All cystic hypersecretory lesions, on microscopy, have cysts filled with an eosinophilic acellular amorphous secretion that resembles thyroid colloid. Secretions frequently retract from the surrounding epithelium, forming scalloped or smooth margins. When cysts are ruptured, the spillage of cystic contents results in an inflammatory response involving lymphocytes and histiocytes.^1^ These cysts are lined by proliferative epithelium, which may be flat at places to papillary fronds. They may show short, knobby epithelial tufts to complex branching fronds, which sometimes extend across the duct lumen.^1^ The cells show intermediate to high-grade nuclear features. CHH is lined by flat, orderly, benign epithelium.^2^ An invasive component produces a distinct solid mass or nodule. It may also present with just thickening of the cyst wall as seen in our case. The invasive component is usually poorly differentiated ductal carcinoma and may be seen microscopically as small clusters or single cells. The focus of invasive carcinoma may show clear and vacuolated nuclei, similar to papillary thyroid carcinoma.^1^

On histological examination, CHC needs to be differentiated from a number of benign intraductal proliferative lesions, juvenile papillomatosis, mucocele-like lesions, mucinous cystadenocarcinoma, secretory carcinoma, pregnancy-like hyperplasia, a micropapillary variant of DCIS as well as cystic metastasis from papillary thyroid carcinoma.^7^ Due to their rarity, these cases are frequently misdiagnosed as benign or misclassified as non-specific ductal carcinoma in situ (DCIS).^1^ A benign lesion such as juvenile papillomatosis is distinguished from cystic hypersecretory carcinoma by its complex histologic features, including foci of sclerosing adenosis, papillary apocrine change, and myoepithelial proliferation. These lesions are more common in children. Generally, the benign disease manifested by microcysts is not associated with colloid-like secretion. Microcystic appearance combined with bland nuclear chromatin may result in a misdiagnosis of fibrocystic disease. Mucocele is a collection of cysts lined by flat or columnar epithelium exhibiting focal hyperplasia. They are distinguished from CHC by their coarse microcalcification and absence of colloid-like secretions. Ductal carcinoma in situ with comedo necrosis is the most likely differential diagnosis. CHC lacks the Roman arch bridging pattern observed in micropapillary DCIS. In micropapillary DCIS, there is a lack of colloid-like secretion in the lumen.^1^^,^^7^ Secretory carcinoma shows multiple microcysts with eosinophilic secretions and vacuolar cytoplasm. While large dilated cysts characterize CHC, cysts in secretory carcinoma have pale blue basophilic secretions, not seen in CHC.^7^ Breast secretory carcinoma is ER-negative and S100 positive, which are characteristic of ETV6-NTRK3 gene fusions. Most CHH and CHC are ER-positive, and in cases where ER is negative high-grade nuclear features are seen as a trait that is not seen in secretory carcinomas.^5^ On reviewing the literature, no molecular alterations have been identified. Metastasis from papillary thyroid carcinoma is also a differential due to the cystic nature, nuclear features, and colloid-like appearance of the secretions. It can be ruled out by proper history taking and appropriate immunohistochemistry (ER, PR, HER2/neu for breast and TTF-1 and thyroglobulin for thyroid).^7^ There was no history of papillary thyroid carcinoma in our patient. Hence, it was excluded.

Prognostic markers exhibit variable immunohistochemical expression. D’Alfonso et al.^5^ reported that four of ten cases were ER and PR positive in a series study. Skalova et al.,^7^ reported three of five cases to be HER2/neu positive while only one case was triple negative. In our case ER, PR were negative, and HER2/neu was positive, which was also noted in few other case reports.^2^^,^^4^^,^^8^

## CONCLUSIONS

Invasive CHC is a rare subtype of breast carcinoma that is characterized by multiple cysts lined by a variable epithelial lining and secretions that are eosinophilic, amorphous, and acellular. As they are rare, there is a lack of information on these lesions, comprising a few case reports or short case series describing clinical, radiological, and morphological characteristics. No large study has been conducted to date, to evaluate the biological behavior, genetic and molecular alterations associated with cystic hypersecretory lesions. The prognosis of these lesions is also not well documented. It is vital for pathologists to be aware of this entity to avoid misdiagnosis, especially as benign lesion on core/ excision biopsy as they are often done clinically so that patients can undergo mastectomy and appropriate treatment.
